# Mixing Ratio and Packaging Amount Synergistically Improved Antioxidant Properties of Baby Lettuce (*Lactuca sativa* L.) and Spinach (*Spinacia oleracea* L.) Mixes

**DOI:** 10.3390/foods15030499

**Published:** 2026-02-01

**Authors:** Lijuan Zhan, Cosimo M. Profico, Giuseppe Pignata, Manuela Casale, Han Gao, Marco Devecchi, Silvana Nicola

**Affiliations:** 1 College of Food Science and Technology, Henan Agricultural University, No. 95 Wenhua Road, Zhengzhou 450002, China; ljzhan@henau.edu.cn (L.Z.); gaoduo397@163.com (H.G.); 2 Department of Agricultural, Forest and Food Sciences, University of Turin, Largo Paolo Braccini, 2, 10095 Grugliasco, TO, Italy; cosimomatteo.profico@unito.it (C.M.P.); giuseppe.pignata@unito.it (G.P.); manuela.casale@unito.it (M.C.); marco.devecchi@unito.it (M.D.)

**Keywords:** fresh-cut vegetables, packaging method, food quality, antioxidants, modified atmosphere packaging

## Abstract

Fresh baby leaves are commercially marketed in various mixing ratios and packaging amounts, creating very distinct microenvironmental conditions that significantly affect the postharvest quality of the fresh product. This study investigated the synergistic effect of mixing ratio (50LB, 50% lettuce + 50% spinach; 75LB, 75% lettuce + 25% spinach; 100LB, 100% lettuce) and packaging amount (125F, 125 g; 250F, 250 g) on the antioxidant qualities of baby lettuce and spinach mixes during 9 days of storage at 4 °C. The results showed that 50LB × 250F inhibited the degradation of chlorophyll and carotenoids and preserved 28% higher total antioxidant capacity (TAC), 43% higher total phenolic compounds (TPC), and 20% higher vitamin C (Vit.C) than the mean values of all samples, resulting in 0.8% lower O_2_ and 14.7% higher CO_2_ levels at the end of storage. TPC, Vit.C, and carotenoids were the main contributors to TAC, with strong correlations (*p <* 0.001). The total bacterial (TB) and yeast + mold (Y + M) counts were only affected by the mixing ratios, with TB increasing by only 1 Log_10_ cfu g^−1^ FW, and Y + M remaining within the same order of magnitude over time. After 9 days of storage, the leaves were still fresh and marketable. This study not only provides a practical strategy for the fresh-cut industry to enhance product quality but also underscores the significance of multifactorial synergism in salad mix packaging.

## 1. Introduction

Baby leaf vegetables (BLVs) are a category of leafy vegetables that are harvested for consumption at the juvenile stage [1,2]. They are often mixed and marketed as salad mixes, which offer consumers diverse tastes, textures, and bioactive compounds [3,4,5]. They currently represent an innovative food category and have shown an increasing market share in the fresh-cut sector [6]. Recent reviews specifically highlight green leafy vegetables as a priority target for shelf-life extension through minimal processing and novel techniques [7]. However, BLVs are highly perishable and susceptible to quality deterioration during the supply chain, as their immature leaves exhibit higher respiration and metabolic activity in comparison to their fully grown counterparts [8,9]. The major challenge facing the BLV industry is rapid quality deterioration and a reduced shelf life. Thus, for all actors in the BLV supply chain, innovative and feasible strategies are urgent [10].

After harvest, BLVs are sold washed or unwashed either in plastic trays or in bags under a modified atmosphere to minimize physical damage and prevent wilting [3]. Modified-atmosphere packaging (MAP) is currently considered an effective and widely implemented strategy to preserve the quality and extend the shelf life of fresh products [11,12]. The packaging amount in a given-size package impacts the free headspace volume, gas concentration, and equilibrium state in packages, which, in turn, influences the product’s quality and shelf life [9]. Generally, the greater the amount of leaf packaging, the faster the modified-atmosphere equilibrium is reached. Such fast modified-atmosphere equilibrium, characterized by decreased O_2_ and increased CO_2_ concentration, is desirable for reducing product respiration and oxidative metabolism, as well as inhibiting the growth of aerobic mesophilic bacteria [3]. However, increasing the amount of leaf packaging reduces the free headspace volume in packages and thus increases the risk of crushing injury and quality deterioration of BLVs during subsequent storage. In addition, an excessive amount of packaging may induce anaerobic fermentation and off-odor development when extremely-low O_2_ and high CO_2_ concentrations occur inside the packaging [3,13,14]. Conversely, a lower packaging amount reduces potential crushing damage but slows gas equilibrium, thereby accelerating respiration and undesirable quality decay of leaves due to prolonged high O_2_ availability in the package [9,15,16]. Therefore, the optimum packaging amount in a given-size package is a critical limiting factor in modifying BLV quality and shelf life.

Apart from packaging amount, the mixing ratio of different types of leaves also significantly influences BLVs’ postharvest behaviors [9]. To meet consumers’ diverse demands, BLVs are often marketed as mixtures containing two or more species [3,9]. Unlike a single fresh commodity, BLV mixtures are complex combinations of different baby leaves with various physiological characteristics, such as respiration rate and tolerance to gas concentration [1,3]. Thus, different mixing ratios of different BLV species or varieties are expected to establish distinct atmospheric scenarios within the package, which, in turn, strongly influence BLVs’ postharvest quality [16]. For example, a mixing ratio consisting of 25% green lettuce and 75% red lettuce resulted in improved visual color (pigments) and inherent quality of baby lettuce mixes compared with other mixing ratios throughout shelf life [9]. Conversely, for green lettuce and rocket mixtures, a mixing ratio of 50% lettuce and 50% rocket salad has been identified as favorable for preserving chlorophyll and internal nutrients during cold storage [9,17]. Hence, the optimal mixing ratio varies depending on vegetable type, underlining the necessity of optimizing mixing ratios for different BLV mixtures.

Lettuce (*Lactuca sativa* L.) and spinach (*Spinacia oleracea* L.) are popular leafy vegetables grown worldwide. They are valuable sources of antioxidant compounds, including carotenoids, total phenolic compounds, vitamin C, and other various health-beneficial bioactive substances [18,19,20,21]. Both baby lettuce and spinach are key components of salad mixes, whose consumption is increasing and has gained popularity as a culinary trend worldwide [22,23]. Commercially, to meet consumers’ diverse demands and increase product attractiveness, lettuce and spinach are often mixed and marketed with different mixing ratios and packaging amounts. This practice creates a micro-atmosphere distinct from that of single-species packages and strongly affects postharvest quality.

Nevertheless, most of the literature has focused on either lettuce or spinach individually when evaluating postharvest behavior influenced by pre- and postharvest factors [21,22,24]. Few studies have evaluated the synergistic effect of mixing ratio and packaging amount on the postharvest fate of these two species when combined. To address this knowledge gap, the aim of this study was to investigate the synergetic effect of mixing ratio and packaging amount on the postharvest quality of lettuce and spinach mixtures during storage.

## 2. Materials and Methods

### 2.1. Plant Materials

The raw materials were obtained from cultivations carried out at the Agricultural and Livestock Experimental Station Tetto Frati (44°53′11.67″ N; 7°41′7.00″ E—231 m a.s.l., Carmagnola, TO, Italy) from October to January. Lettuce (*Lactuca sativa* L. var. *crispa* cv. Lollo Bionda, hereafter: LB) and spinach (*Spinacia oleracea* L. F1 Auriga SRX 1500, hereafter: SA) were seeded in 60-cell Styrofoam trays (ca. 784 and ca. 1176 plants/m^2^, respectively) filled with a commercial peat-based horticultural medium. The BLVs were grown in a greenhouse in a lab-scale pilot plant (LSPP), using a continuous floating growing system (FGS, plants grow on floating trays in tanks or water beds with nutrient solution) with a 40/60 N-NO_3_^−^/N-NH_4_^+^ hydroponic nutrient solution (HNS) composed of (all in mmol L^−1^): 6 N, 2 P, 6 K, 2 Mg, and 2.5 Ca. The nutrient solution had an electrical conductivity (EC) of 0.2 dS m^−1^ and the pH was monitored and regularly maintained at 5.5 using H_2_SO_4_. The greenhouse was equipped with an automatically controlled temperature system. During the growing season, the maximum, minimum, and mean temperatures were 20 °C, 10 °C, and 15 °C, respectively, with a relative humidity of 82%. A randomized complete block design (RCBD) was used for the experiment. The treatments were arranged in a single factorial design comprising two species and three blocks during the raw material production. At harvest (75 days after seeding), the leaves were immediately transferred to the postharvest laboratory located at the same site as the LSPP for processing.

### 2.2. Samples Preparation

**Figure 1:**
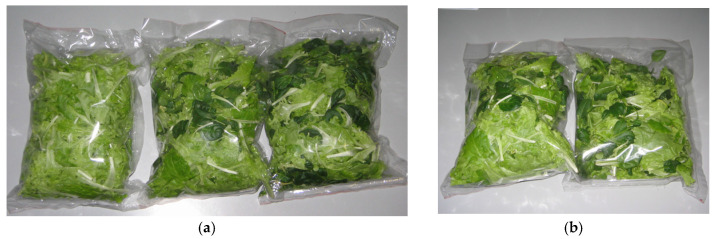
Commercial mixing ratios and packaging amounts: representation of the treatments. (**a**): Three mixing ratios: 100LB, 75LB, 50LB. (**b**): Two packaging amounts: 250F, 125F.

The raw material was sorted in a cold room and any damaged and yellow leaves were discarded. Three weight-based mixing ratios, 50LB (50% lettuce + 50% spinach), 75LB (75% lettuce + 25% spinach), and 100LB (100% lettuce), were prepared, respectively. The mixtures were subsequently packaged with two packaging weights of 125F (125 g) and 250F (250 g) in 0.25 m × 0.35 m plastic bags before heat sealing (Figure 1). The bags were previously prepared using polypropylene film with a thickness of 20 µm, weight of 18.2 g m^−2^, permeability to oxygen and carbon dioxide of 1990 and 7800 cm^3^ m^−2^ d^−1^ bar^−1^, respectively, and water vapor permeability of 5.8 gm^−2^ d^−1^ bar^−1^ (Alvapack S.r.l., Bologna, Italy). A total of 36 samples (3 mixing ratios × 2 packaging amounts × 3 blocks × 2 sampling days) were packaged according to the experimental design. The packaged samples were stored at 4 °C for a 9-day shelf life in refrigerated chambers without light. Parameter analyses were conducted at harvest (d0), after 1 day (d1), and after 9 days (d9) of storage. Microorganism counts were assayed only at d0 and d9.

### 2.3. Headspace Gas Composition and Microbial Assay

The headspace gas (O_2_% and CO_2_%) content was measured using a Check Point Handheld Gas Analyzer (PBI-Dansensor AS, Ringsted, Denmark). Prior to the assay, the instrument was calibrated according to the manufacturer’s instructions: air was used for O_2_ calibration, and 100% CO_2_ and pure nitrogen were used for CO_2_ calibration. The total bacterial (TB) and the yeast + mold (Y + M) counts were determined according to international standards [25,26] using Plate Count Agar (Fluka Analytical, Sigma-Aldrich S.r.l., Milan, Italy) and Yeast Extract Glucose Chloramphenicol Agar (Fluka Analytical, Sigma-Aldrich S.r.l., Milan, Italy), respectively, on 25.0 g of fresh tissue after incubation for 48 h at 30 °C.

### 2.4. Chlorophyll, Carotenoids, Fresh Weight Loss (FWL), and Dry Matter (DM) Analysis

The chlorophyll a (*Chl a*), chlorophyll b (*Chl b*), and carotenoid contents were determined according to the Lichtenthaler and Wellburn (1985) [27] method. Briefly, 1 g plant tissue was ground in 5 mL 80% acetone/water (*v*/*v*) solution and extracted at 4 °C in darkness overnight. The extract was centrifuged at 15,000× *g* at 4 °C for 20 min. Subsequently, the absorbance of the centrifuged supernatant was measured at 662, 645, and 470 nm, respectively. The quantities of *Chl a*, *Chl b*, and carotenoids were calculated according to the Lichtenthaler and Wellburn Formulas (1)–(4) [27].

(1) C_a_ = 11.75 A_662_ − 2.35 A_645_

(2) C_b_ = 18.61 A_645_ − 3.96 A_662_

(3) C_t_ = C_a_ + C_b_

(4) C_x+c_ = 1000 A_470_ − 2.27 C_a_ − 81.4 C_b_/227

where C_a_ = chlorophyll a (mg L^−1^); C_b_ = chlorophyll b (mg L^−1^); C_t_ = total chlorophyll (mg L^−1^); and C_x+c_ = total carotenoids (mg L^−1^).

FWL was measured by weighing the samples daily during storage and calculated by comparing sample weights over the storage period. The results were expressed as percentages. DM was measured by drying fresh plants in an oven at 60 °C until constant weight and calculated as the percentage of constant weight relative to fresh-plant weight.

### 2.5. TAC and Phytochemical Compounds Assay

The standard curves of total antioxidant capacity, total phenolic compounds, and vitamin C are included in the Supplementary Materials (Figure S1).

#### 2.5.1. TAC Assay

One gram of frozen tissue was homogenized in 5 mL pure methanol and the homogenates were centrifuged at 15,000× *g* at 4 °C for 20 min. TAC was evaluated by ferric-reducing antioxidant power (FRAP) assay [28]. The fresh FRAP reagent was prepared by mixing 300 mM sodium acetate-acetic acid buffer (pH 3.6), 20 mM FeCl_3_, and 10 mM TPTZ (2,4,6-tripyridyl-s-stiazine) in a 10:1:1 proportion. The analysis was carried out by mixing 980 µL fresh FRAP reagent and 20 µL aliquot of methanol extract. The mixture was incubated at 20 °C for 4 min before absorbance at 593 nm wavelength was determined. TAC values were calculated according to the standard curve, prepared using fresh ammonium ferrous sulfate, and expressed as micromole ferrous ion per g of fresh weight (µmol Fe^2+^ g^−1^ FW).

#### 2.5.2. TPC Assay

The supernatant was collected for the TPC assay. TPC was measured using the Folin–Ciocalteu procedure [29] with some modifications. The 0.1 mL aliquot of the supernatant prepared in Section 2.5.1 was mixed in 0.5 mL Folin–Ciocalteu reagent. After standing for 3 min, 0.4 mL of 7.5% NaCO_3_ was added. The contents of the tubes were thoroughly mixed and then incubated for 30 min at 20 °C. The absorbance of the mixture was read at 760 nm, and the TPC content was calculated according to the standard curve produced by gallic acid.

#### 2.5.3. Vit.C Assay

The Vit.C content was determined spectrophotometrically according to the method of Kampfenkel et al. (1995) [30] with some modifications. One gram of leaves was ground in 3.5 mL of 6% freezing trichloroacetic acid (TCA)/water (*w*/*v*) on ice. The homogenate was centrifuged at 15,000× *g* at 4 °C for 10 min, and the supernatant was immediately used for the Vit.C analysis. For each measurement, a 0.2 mL supernatant was combined with 0.2 mL of 10 mM dithiothreitol (DDT) in 0.4 mL of 0.2 M phosphate-buffered solution (pH 7.4). After incubation for 15 min at 42 °C, the mixture was supplemented with 0.2 mL of 0.5% N-ethylmaleimide (NEM) and incubated for 1 min at room temperature. Subsequently, 1.0 mL of 10% TCA, 0.8 mL of 42% H_3_PO_4_, 0.8 mL of 4% 2,2′-dipyridyl ethanol solution, and 0.4 mL of 3% FeCl_3_ were added. The mixture was immediately vortexed vigorously and incubated for 40 min at 42 °C to allow color development. The absorbance was read at 525 nm. Results were expressed as milligram per gram of fresh weight (mg g^−1^ FW) based on calibrations that were compared with standard curves produced using freshly prepared L-ascorbic acid.

### 2.6. Statistical Analysis

The data were submitted for analysis of variance (ANOVA) using the Statistical Package for Social Science (IBM SPSS Statistics, Version 31.0, IBM Corp., Armonk, NY, USA) considering species, mixing ratio, packaging amount, and their interaction as the variation factors at each storage time point. When ANOVA was significant, species and packaging amount effects were tested using the F-test, and the effects of mixing ratio and the interaction between mixing ratio × packaging amount were tested using Tukey’s multiple-comparison test. Spearman’s correlation analysis was used to test the bivariate correlations, which were considered statistically significant with a two-tailed *p* value less than 0.05.

## 3. Results

### 3.1. Headspace Gas Composition and Microbial Count

The contents of O_2_ and CO_2_ were significantly influenced by both mixing ratio and packaging amount, but not by their interaction, at d1 and d9 of storage (Table 1). Treatment 100LB resulted in the highest O_2_ (13.58%) and lowest CO_2_ (5.00%) contents, whereas treatment 50LB induced the lowest O_2_ (3.15%) and highest CO_2_ (12.53%) contents at d9 of storage. Thus, the increase in spinach mixing ratio significantly increased respiration and quickly created a modified-atmosphere equilibrium in the packages. As expected, the 250F package had significantly lower O_2_ (7.36% vs. 10.31%) and higher CO_2_ (10.59% vs. 7.16%) concentrations than the 125F package during storage (Table 1), indicating that a higher packaging amount could accelerate the modified-atmosphere equilibrium in packaging by consuming more O_2_ and producing more CO_2_. The 50LB × 250F treatment resulted in the lowest O_2_% (0.80%) and highest CO_2_% (14.70%) among all interaction samples, although no significant difference was observed due to the high standard error.

The TB and Y + M counts were significantly influenced by species at harvest (Table 2). The amounts of TB and Y + M in spinach leaves were 2.51- and 4.48-fold higher, respectively, than those in lettuce leaves at harvest. The average amounts of TB and Y + M in lettuce and spinach raw tissues were 4.20 and 1.78 Log_10_ cfu g^−1^ FW, respectively. During storage, both the TB and Y + M counts were only significantly influenced by the mixing ratio treatment (Table 1). The counts of TB and Y + M in treatments 75LB and 50LB were statistically higher than those in the 100LB treatment, while no significant difference was observed between the 75LB and 50 LB treatment. After 9 d of storage, the mean TB count showed an increase of about one Log_10_ cfu g^−1^ FW (4.20 vs. 5.33 Log_10_ cfu g^−1^ FW), and the mean Y + M count remained within the same order of magnitude (1.78 vs. 1.65 Log_10_ cfu g^−1^ FW).

### 3.2. Chlorophyll Content and Fresh Weight Loss

At harvest, *Chl b* content was significantly affected by species with an 80% higher content in spinach than in lettuce (Table 2). After harvest, both *Chl a* and *Chl b* contents were significantly influenced by mixing ratio and the interaction of mixing ratio × packaging amount during the whole storage period (Table 3). Treatment 50LB × 250F resulted in the highest, while 100LB × 250F had the lowest values of both *Chl a* and *Chl b* concentrations at the end of storage. In detail, *Chl a* and *Chl b* contents in samples of 50LB × 250F were 164% and 62% higher than those in samples of 100LB × 250F at the end of storage, respectively. In terms of mixing ratio, treatment 50LB showed significantly higher *Chl a* and *Chl b* contents than 75LB, which in turn was significantly higher than 100LB at the end of storage. In summary, the 50LB mix preserved the highest *Chl a* and *Chl b* contents, and 100LB retained the lowest at d9 storage (Table 3). In addition, the mean values of both *Chl a* and *Chl b* decreased over time, with *Chl a* decreasing more slowly than *Chl b* (14.28% vs. 26.67%), resulting in an increasing trend in the *Chl a/b* ratio regardless of treatment (Table 3). This observation is consistent with the chlorophyll catabolism model, during which *Chl b* must be converted into *Chl a* to enter the degradation pathway, thereby resulting in an apparent retardation of *Chl a* degradation. The FWL was not significantly affected by any main factor and their interaction, with the average values of all samples being 0.37% at d9 storage (Table S1 in the Supplementary Materials). It significantly increased over shelf life (*p <* 0.01). The maximum FWL reached 0.49%, which occurred in samples of 100LB × 125F at the end of storage (Table S1), and was much lower than the threshold of 2% fresh weight loss for unacceptable leafy vegetables, indicating that all leaves were still turgid and marketable after 9 days of storage at 4 °C.

### 3.3. TAC and Phytochemical Compounds

#### 3.3.1. TAC

At harvest, there was a significant difference in TAC values between spinach and lettuce raw leaves; the former was 71.25% higher than the latter (Table 2). After harvest, TAC values were only significantly influenced by mixing ratio at both d1 and d9 (Table 4). The 50LB treatment resulted in significantly higher TAC values than 75LB and 100LB over time; the latter two were statistically similar in TAC values at d1. At d9, the TAC values of 50LB were significantly higher than those of 75LB, and those of 75LB were significantly higher than those of 100LB, indicating that TAC values increased with increasing spinach proportion. It is worth noting that the mean TAC values remained constant from d1 (11.61 µmol Fe^2+^ g^−1^ FW) to d9 (11.91 µmol Fe^2+^ g^−1^ FW) of storage, both of which were clearly higher than the average TAC values of the raw material at harvest (9.67 µmol Fe^2+^ g^−1^ FW) (Table 2). This indicates that the postharvest practices adopted in this study were favorable for maintaining or even increasing TAC values of baby spinach and lettuce mixes during storage.

#### 3.3.2. TPC

At harvest, the TPC content of the spinach was 1.59-fold higher than that of lettuce, although no significant difference was observed (*p =* 0.062) (Table 2). During storage, TPC content was significantly influenced by mixing ratio and the interaction of mixing ratio × packaging amount at d1 and by mixing ratio only at d9 (Table 4). The 50LB × 250F treatment resulted in the highest TPC content, while the 75LB × 250F had the lowest TPC content at d1. At the end of storage, 50LB and 75LB resulted in similar TPC contents, both of which were significantly higher than 100LB (Table 4). Similarly to TAC evolution, the average TPC values remained constant from d1 (0.47 mg gallic acid g^−1^ FW) to d9 (0.48 mg gallic acid g^−1^ FW) of storage, indicating that all leaves were rich in TPC after 9 days of shelf life.

#### 3.3.3. Vit.C

At harvest, the Vit.C content of the spinach was 1.77 times higher than that in lettuce, with an average value of 0.43 mg g^−1^ FW; however, no significant difference was observed due to the high standard error (Table 2). Upon storage, Vit.C content was only significantly affected by mixing ratio at both d1 and d9 (Table 4). The highest Vit.C content was observed in leaves from 50LB, which was more than twice that in leaves from 75LB at d1. At d9, Vit.C content in 50LB samples was the same as that in 75LB samples, but significantly higher than that in 100LB samples, and no significant difference existed between the latter two (Table 4). Interestingly, compared with the average Vit.C value at harvest (0.43 mg g^−1^ FW), the average Vit.C content of all leaves increased during postharvest storage, reaching 0.61 mg g^−1^ FW after 1 day and 0.65 mg g^−1^ FW after 9 days of storage (Table 2 and Table 4).

#### 3.3.4. Carotenoids

Carotenoid content was not significantly influenced by species at harvest, with an average value of 0.08 mg g^−1^ FW (Table 2). It was significantly affected by the two main factors at d1 and by the two main factors and their interaction at d9 (Table 4). Treatments of 75LB resulted in the same carotenoid content as 50LB, but significantly higher than 100LB; the latter two had statistically similar carotenoid content at d1. After 9 days of storage, carotenoids content in the 50LB sample showed a slight increase and was significantly higher than that of 75LB and 100LB; both of the latter decreased over time, and 75LB remained significantly higher than 100LB (Table 4). In terms of packaging amount, the 250F treatment preserved significantly more carotenoids than 125F at d1 storage. However, the opposite trend was observed at d9, with leaves from the 125F treatment containing more carotenoids than those from 250F (Table 4). At the end of storage, the combination of 50LB × 250F resulted in the highest carotenoid content (0.11 mg g^−1^ FW), which was 2.20-fold higher than the lowest value (0.05 mg g^−1^ FW) detected in 100LB × 250F samples. The mean carotenoid content across treatments was 0.08 mg g^−1^ FW at d9, the same as the average values of the raw spinach and lettuce at harvest, indicating that carotenoid levels remained constant during storage.

## 4. Discussion

Efforts are undertaken during the BLV supply chain to obtain high-quality raw materials at harvest and minimize quality deterioration between harvest and consumption. In recent years, innovative soilless culture systems, such as FGS, have been established to cultivate BLVs with high yield, quality, and safety at harvest [31,32]. As expected, the FGS adopted in this study produced baby lettuce and spinach with high fresh yields (lettuce 1971.89 g m^−2^; spinach 1217.18 g m^−2^) and high levels of phytochemical compounds, such as Vit.C, total polyphenols, carotenoids, and chlorophyll, prior to storage (Table 2). This is in accordance with previous conclusions that FGS is suitable for producing leafy vegetables with higher qualitative and quantitative yields than traditional cultivation techniques [2,33]. In particular, in this study, FGS produced spinach with remarkably higher Vit.C (0.55 mg g^−1^ FW vs. not detected) and dry matter (DM) content (12.53% vs. 10.0%) (Table 2) than open-field soil cultivation during the same growing cycle (sowing in October and harvest in January) in Italy [34]. This is possibly the result of the greater availability of nutrients in FGS and warmer environmental conditions in the greenhouse (ca. 20/10 °C day/night temperature in our greenhouse during the material growth period) compared with open-field conditions, where below- or near-zero temperatures often occur during the December–January period in Italy. Such near-freezing temperatures can damage cell membranes and reduce phytochemical accumulation [24,34,35].

The postharvest safety of BLVs as salad mixes in the fresh-cut sector has been extensively investigated in terms of microbial growth. However, the original microbial population of BLV raw materials at exactly harvest time has been much less documented. Most studies concerning postharvest BLV microbial contamination have acquired raw material either from fresh markets, with unknown lag time between harvest and analysis, or from commercial farms, with subsequent washing procedures and microbial incubation to investigate the influence of postharvest operations (e.g., washing) on microbial load [32,36]. In both cases, the original microbial contamination of raw materials at harvest was not accurately represented [32]. Most reports show that microbial contamination of ready-to-eat salads ranges between 2.0 and 9.0 Log_10_ cfu g^−1^ FW for total aerobic colony counts and 2.9–6.5 Log_10_ cfu g^−1^ FW for yeasts, respectively [37]. In the present study, the average TB and Y + M counts were 4.20 Log_10_ cfu g^−1^ FW and 1.78 Log_10_ cfu g^−1^ FW at harvest, respectively (Table 2). These values were lower than those reported for ready-to-eat salad raw materials from industry and supermarkets in Italy, which exhibited total aerobic colony counts of 6.3–6.7 Log_10_ cfu g^−1^ FW and yeasts + mold counts of 1.8–4.2 Log_10_ cfu g^−1^ FW before washing [37], confirming that FGS is an innovative and practicable alternative for producing clean raw BLVs at harvest for the fresh-cut sector. Furthermore, TB and Y + M counts increased only slightly (TB means of 4.20 Log_10_ cfu g^−1^ FW at harvest and 5.33 Log_10_ cfu g^−1^ FW at d9; Y + M means of 1.78 Log_10_ cfu g^−1^ FW at harvest and 1.65 Log_10_ cfu g^−1^ FW at d9) during 9 d of storage regardless of treatment (Table 1 and Table 2). This indicates that the combination of preharvest FGS and postharvest strategies adopted in this study had a synergistic effect in inhibiting microbial reproduction. Such slow microbial growth can be explained by two main factors: minimized mechanical damage and the maintenance of consistently low storage temperatures. As is well known, fresh vegetables are perishable and provide favorable substrates for microbial proliferation, particularly when mechanical injury occurs during processing, which creates entry points and increases nutrient availability, thereby accelerating spoilage [38]. In this study, BLV leaves were not subjected to further cutting and washing procedures that cause irreversible physical damage after harvest. During storage, all leaves were packaged and placed on chamber shelves in a single layer, with much less handling than commercial products, which often experience stacking and frequent turning during transport and display. This reduced movement minimized the potential for compression and collision injury. The only mechanical damage occurred during petiole cutting at harvest. Such reduced mechanical injury helps preserve leaf tissue integrity and reduce juice leakage, thereby limiting nutrient availability for microbial growth. In addition to physical integrity, temperature control played a critical role. Samples were stored at a constant 4 °C throughout storage, without temperature abuse or fluctuations that often occur during commercial transport and retail display. This continuous low storage temperature effectively inhibited microbial metabolic and enzymatic activity. For instance, consistent refrigeration at 4 °C is known to markedly slow the growth of total aerobic bacteria, yeasts, and molds on ready-to-eat spinach, whereas growth rates increase substantially at temperatures of 8 °C and above [39]. Similar results were reported by Calonico et al. (2019) [37], who observed that yeast and mold counts remained almost unchanged during refrigeration at approximately 4 °C for 7 days, probably due to their reduced capacity for growth at low temperatures.

It is worth noting that spinach exhibited significantly higher microbial contamination than lettuce in both TB (6.01 vs. 2.39 Log_10_ cfu g^−1^ FW) and Y + M (2.91 vs. 0.65 Log_10_ cfu g^−1^ FW) counts at harvest, although both were grown under the same conditions (Table 2). This difference could be attributed to the thinner and shorter petioles of spinach compared with lettuce, which allow spinach globular leaves to remain closer to the surface of the culture medium, thereby increasing the risk of contamination [32]. In addition, the higher microbial load observed on spinach leaves may be related to the presence of vermiculite on the surface of the culture medium, according to our further observations (data not published). Therefore, to reduce microbial contamination, the use of spinach varieties with erect and sturdy petioles, as well as vermiculite-free culture media, is suggested for FGS cultivation.

**Figure 2:**
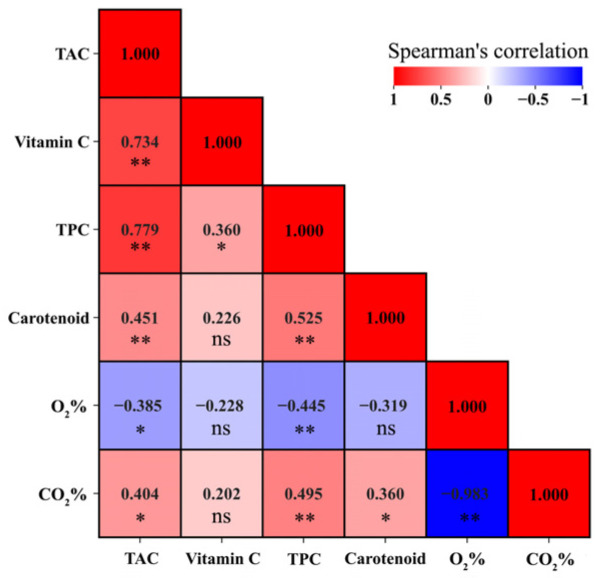
Spearman’s correlations analysis among select variables. TAC, total antioxidant capacity; TPC, total phenolic compounds; The correlation coefficients are proportional to the color intensity. Positive correlation is displayed in red and negative in blue. * means the is correlation significant at the 0.05 level (2-tailed); ** means the correlation is significant at the 0.01 level (2-tailed); ns means not significant. Total of 36 replicates for each parameter.

The mixing ratio and packaging amount adopted in this study showed a synergetic effect on BLV quality evolution, with 50LB × 250F preserving significantly higher chlorophyll content during storage (Table 3), higher total phenolic content at d1, and higher carotenoid content at d9 (Table 4). Meanwhile, 50LB × 250F samples also exhibited 28% higher TAC, 43% higher TPC, and 20% higher Vit.C than the mean values of all samples at the end of storage, although no significant differences were detected among treatments (Table 4). Considering the possible concentration of compounds due to moisture loss, fresh weight loss (mainly moisture loss) was monitored throughout storage and ranged from 0.22% to 0.49% (Supplementary Table S1). After recalculating the data to eliminate this concentration effect, TAC, TPC, and Vit.C levels remained unchanged, confirming that the observed increases reflected genuine changes in phytochemical content. Such beneficial interactive effects may be attributed to the combined contribution of high initial raw material quality at harvest and favorable modified atmospheres characterized by lower O_2_ (0.80%) and higher CO_2_ (14.70%) levels during storage. As shown in Table 2, spinach exhibited numerically higher pigment and phytochemical content than lettuce at harvest. Thus, increasing the proportion of spinach (50BL) contributed to the superior quality of 50LB × 250F samples. In addition, the higher packaging amount (250F) in a given package size promoted rapid gas equilibrium, resulting in low-O_2_ (0.80%) and high-CO_2_ (14.70%) conditions within the 50LB × 250F sample headspace, which are favorable for limiting oxidative degradation of pigments and phytochemicals through respiration inhibition. This interpretation is supported by the observed negative correlations between these phytochemicals and O_2_ content, as well as positive correlations with CO_2_ content (Figure 2). Similar findings have been reported, showing that low-O_2_ controlled atmospheres are beneficial for maintaining respiration rates and total antioxidant capacity in spinach and kale [40,41]. Furthermore, unlike active modified-atmosphere packaging, in which the desired atmosphere is rapidly established by gas flushing, the modified atmosphere in 50LB × 250F packages in this study was passively and progressively established during storage. This resulted in a dynamic microenvironment with gradually decreasing O_2_ and increasing CO_2_ levels, thereby avoiding potential gas injury associated with prolonged exposure to extremely low O_2_ (<0.4%) and high CO_2_ (>13%) concentrations [42]. Finally, as expected, antioxidant quality in mixed lettuce and spinach was mainly attributed to TPC, Vit.C, and carotenoids, as indicated by the strong positive correlations (*p <* 0.001) between these bioactive substances and TAC values (Figure 2). This observation is fully consistent with previous findings identifying TPC and Vit.C as major contributors to TAC in spinach [43].

## 5. Conclusions

This study demonstrates that the floating growing system (FGS) is a feasible approach for producing baby leaf vegetables with high quality and low microbial contamination at harvest. The combined effects of mixing ratio and packaging amount significantly influenced postharvest quality, with the 50LB × 250F treatment showing the most favorable outcomes in terms of chlorophyll retention and phytochemical preservation under low-O_2_ and high-CO_2_ conditions. High levels of total phenolic compounds, vitamin C, and carotenoids were maintained and were the main contributors to antioxidant capacity. However, the superior quality observed was partly attributable to the higher proportion of spinach, highlighting its role as a confounding factor in interpreting synergistic effects. Fresh weight loss remained minimal, and microbial growth was limited during storage, indicating good product stability. Further research should focus on optimizing packaging materials and gas permeability and on assessing foodborne pathogen risks to further improve the safety and quality of mixed baby leaf salads.

## Figures and Tables

**Table 1:** Bag headspace gas composition and microbial count of lettuce and spinach during 9 d storage at 4 °C.

	O_2_ (%)				CO_2_ (%)				TB (Log_10_ cfu g^−1^ FW)		Y + M (Log_10_ cfu g^−1^ FW)	
	d1		d9		d1		d9		d9		d9	
Mixing ratios												
100LB	16.93	a	13.58	a	2.77	b	5.00	c	4.29	b	1.00	b
75LB	15.05	b	9.77	b	4.27	a	9.08	b	5.60	a	1.84	a
50LB	15.07	b	3.15	c	4.47	a	12.53	a	6.11	a	2.13	a
Packaging amount												
250F	15.14	b	7.36	b	4.34	a	10.59	a	5.18		1.75	
125F	16.22	a	10.31	a	3.32	b	7.16	b	5.48		1.58	
Mixing ratios × Packaging amount												
100LB × 250F	16.33		12.27		3.30		6.00		3.97		1.25	
100LB × 125F	17.53		14.90		2.23		4.00		4.62		0.74	
75LB × 250F	14.33		9.00		4.87		11.07		5.58		1.83	
75LB × 125 F	15.77		10.53		3.67		7.10		5.62		1.84	
50LB × 250F	14.77		0.80		4.87		14.70		6.00		2.17	
50LB × 125F	15.37		5.50		4.07		10.37		6.21		2.09	
Mean	15.68		8.83		3.83		8.87		5.33		1.65	
SE	0.25		1.21		0.20		0.94		0.21		0.28	
Significance												
Mixing ratios	***		***		***		***		***		**	
Packaging amount	***		**		***		***		ns		ns	
Mixing ratios × Packaging amount	ns		ns		ns		ns		ns		ns	

Different letters within columns indicate significant differences (*p* ≤ 0.05) among the treatments. ** *p* ≤ 0.01, *** *p* ≤ 0.001; ns—not significant; TB—total bacterial; Y + M—yeast + mold; 100LB—100% lettuce; 75LB—75% lettuce + 25% spinach; 50LB—50% lettuce + 50% spinach; 250F—250 g; 125F—125 g; SE—standard error.

**Table 2:** Biological yield, nutritional parameters, and microbial count of lettuce and spinach at harvest.

	Parameters	Yield (g Fresh Mass/m^2^)	DM (%)	TAC (µmol Fe^2+^ g^−1^ FW)	TPC (mg gallic acid g^−1^ FW)	Vit.C (mg g^−1^ FW)	Carotenoids (mg g^−1^ FW)	Chl a (mg g^−1^ FW)	Chl b (mg g^−1^ FW)	TB (Log_10_ cfu g^−1^ FW)	Y + M (Log_10_ cfu g^−1^ FW)
Species											
Lettuce		1971.89 a	5.47 b	7.13 b	0.41	0.31	0.09	0.19	0.10 b	2.39 b	0.65 b
Spinach		1217.18 b	12.53 a	12.21 a	0.65	0.55	0.07	0.21	0.18 a	6.01 a	2.91 a
Mean		1594.53	9.00	9.67	0.53	0.43	0.08	0.20	0.14	4.20	1.78
Significance		***	**	***	ns	ns	ns	ns	*	***	***

Different letters within columns indicate significant differences (*p* ≤ 0.05) among the treatments. * *p* ≤ 0.05, ** *p* ≤ 0.01, *** *p* ≤ 0.001; ns—not significant; DM—dry matter; TAC—total antioxidant capacity; TPC—total phenolic compounds; Vit.C—vitamin C; Chl a—Chlorophyll a, Chl b—Chlorophyll b; TB—total bacterial; Y + M—yeast + mold.

**Table 3:** Chlorophyll content and Chl a/b of lettuce and spinach mixes during 9 d storage at 4 °C.

Treatments	Chl a (mg g^−1^ FW)				Chl b (mg g^−1^ FW)				Chl a/b			
	d1		d9		d1		d9		d1		d9	
Mixing ratios												
100LB	0.19	b	0.13	c	0.15	b	0.09	c	1.27		1.44	
75LB	0.29	a	0.21	b	0.23	a	0.16	b	1.26		1.31	
50LB	0.25	ab	0.27	a	0.19	ab	0.19	a	1.32		1.42	
Packaging amount												
250F	0.26		0.20		0.21		0.15		1.24		1.33	
125F	0.23		0.21		0.17		0.15		1.35		1.40	
Mixing ratios × Packaging amount												
100LB × 250F	0.18		0.11		0.14		0.08		1.29		1.38	
100LB × 125F	0.20		0.16		0.15		0.11		1.33		1.45	
75LB × 250F	0.36		0.20		0.29		0.15		1.24		1.33	
75LB × 125F	0.23		0.23		0.17		0.18		1.35		1.28	
50LB × 250F	0.25		0.29		0.18		0.21		1.39		1.38	
50LB × 125F	0.26		0.25		0.20		0.17		1.30		1.47	
Mean	0.24		0.21		0.19		0.15		1.26		1.40	
SE	0.03		0.01		0.03		0.01		0.04		0.06	
Significance												
Mixing ratio	**		***		*		***		ns		ns	
Packaging amount	ns		ns		ns		ns		ns		ns	
Mixing ratios × Packaging amount	*		**		*		***		ns		ns	

Different letters within columns indicate significant differences (*p* ≤ 0.05) among the treatments. * *p* ≤ 0.05, ** *p* ≤ 0.01, *** *p* ≤ 0.001; ns—not significant; Chl a—Chlorophyll a, Chl b—Chlorophyll b; Chl a/b—Chlorophyll a/Chlorophyll b; 100LB—100% lettuce; 75LB—75% lettuce + 25% spinach; 50LB—50% lettuce + 50% spinach; 250F—250 g; 125F—125 g; SE—standard error.

**Table 4:** Total antioxidant capacity and phytochemical compounds of lettuce and spinach mixes during 9 d storage at 4 °C.

Treatments	TAC (µmol Fe^2+^ g^−1^ FW)				TPC (mg gallic acid g^−1^ FW)				Vit.C (mg g^−1^ FW)				Carotenoids (mg g^−1^ FW)			
	d1		d9		d1		d9		d1		d9		d1		d9	
Mixing ratios																
100LB	10.62	b	7.51	c	0.37	b	0.22	b	0.62	ab	0.56	b	0.08	b	0.06	c
75LB	9.46	b	12.97	b	0.41	b	0.58	a	0.40	b	0.62	ab	0.11	a	0.09	b
50LB	14.76	a	15.27	a	0.63	a	0.65	a	0.81	a	0.77	a	0.09	ab	0.10	a
Packaging amount																
250F	10.77		11.42		0.45		0.48		0.56		0.61		0.10	a	0.08	b
125F	12.46		12.41		0.49		0.49		0.67		0.69		0.09	b	0.09	a
Mixing ratios × packaging amount																
100LB × 250F	10.46		6.80		0.37		0.18		0.60		0.48		0.08		0.05	
100LB × 125F	10.77		8.21		0.37		0.27		0.65		0.64		0.08		0.07	
75LB × 250F	7.13		12.25		0.30		0.57		0.25		0.58		0.12		0.08	
75LB × 125 F	11.80		13.69		0.52		0.58		0.55		0.67		0.09		0.10	
50LB × 250F	14.71		15.22		0.68		0.69		0.82		0.78		0.10		0.11	
50LB × 125F	14.81		15.32		0.58		0.61		0.80		0.77		0.09		0.10	
Mean	11.61		11.91		0.47		0.48		0.61		0.65		0.09		0.08	
SE	1.24		0.57		0.04		0.05		0.11		0.07		0.01		0.004	
Significance																
Mixing ratio	***		***		***		***		**		*		**		***	
Packaging amount	ns		ns		ns		ns		ns		ns		*		*	
Mixing ratios × Packaging amount	ns		ns		**		ns		ns		ns		ns		**	

Different letters within columns indicate significant differences (*p* ≤ 0.05) among the treatments. * *p* ≤ 0.05, ** *p* ≤ 0.01, *** *p* ≤ 0.001; ns—not significant; TAC—total antioxidant capacity; Vit.C—vitamin C; TPC—total phenolic compounds; 100LB—100% lettuce; 75LB—75% lettuce + 25% spinach; 50LB—50% lettuce + 50% spinach; 250F—250 g; 125F—125 g; SE—standard error.

## Data Availability

The original contributions presented in the study are included in the article/Supplementary Materials. Further inquiries can be directed to the corresponding author.

## Supplementary Materials

The following supporting information can be downloaded at https://www.mdpi.com/article/10.3390/foods15030499/s1. Figure S1. Standard curves of total antioxidant capacity, total phenolic compounds, and vitamin C. Table S1: Fresh weigh loss of lettuce and spinach mixes after 9 d storage at 4 °C.

## References

[1] Artés-Hernández F., Castillejo N., Martínez-Zamora L. (2022). UV and visible spectrum LED lighting as abiotic elicitors of bioactive compounds in sprouts, microgreens, and baby leaves—A comprehensive review including their mode of action. Foods.

[2] Johnson M.A., Thakur S. (2025). Sprouts, microgreens, and baby leaves cultivation in controlled environment agriculture- a panacea for global food and nutritional security. Food Biosci..

[3] Gil M.I., Garrido Y., Gil M.I., Beaudry R. (2020). Leafy vegetables: Baby leaves. Controlled and Modified Atmospheres for Fresh and Fresh-Cut Produce.

[4] Koukounaras A., Bantis F., Karatolos N., Melissas C., Vezyroglou A. (2020). Influence of pre-harvest factors on postharvest quality of fresh-cut and baby leafy vegetables. Agronomy.

[5] Nicola S., Cocetta G., Ferrante A., Ertani A., Florkowski W.J., Banks N.H., Shewfelt R.L., Prussia S.E. (2022). Chapter 7—Fresh-cut produce quality: Implications for postharvest. Postharvest Handling.

[6] Remize F., Garcia C. (2024). Fresh-cut vegetables and fruits: Do they really meet sustainability and nutritional benefits?. Curr. Food Sci. Technol. Rep..

[7] Goel R., Kaur D., Kaur R., Younis K., Qadri O.S. (2025). Shelf-life extension of green leafy vegetables through minimal processing: Special emphasis on the use of novel techniques. J. Agr. Food Res..

[8] Mudau A.R., Araya H.T., Mudau F.N. (2019). The quality of baby spinach as affected by developmental stage as well as postharvest storage conditions. Acta Agric. Scand. Sect. B Soil Plant Sci..

[9] Zhan L., Bulgari R., Pignata G., Casale M., Nicola S. (2022). The mixing ratio and filling-amount affect the tissue browning and antioxidant properties of fresh-cut baby leaf lettuce (*Lactuca sativa* L.) and rocket (*Eruca sativa* Mill.) grown in floating growing systems. Foods.

[10] Rocha D.C., Kochi L.Y., Kitamura R.S.A., Brito J.C.M., Nogueira K.D.S., Gomes M.P. (2024). Unveiling the impact of antimicrobial-infused water on hydroponic baby leafy vegetables (lettuce, rocket, and watercress): Physiological effects and food safety. J. Environ. Chem. Eng..

[11] Habiba U., Bajpai A., Shafi Z., Pandey V.K., Singh R. (2025). Advancing sustainability through modified atmosphere packaging (MAP) for fresh food preservation: A critical review. J. Stored Prod. Res..

[12] Ghidelli C., Pérez-Gago M.B. (2018). Recent advances in modified atmosphere packaging and edible coatings to maintain quality of fresh-cut fruits and vegetables. Crit. Rev. Food Sci. Nutr..

[13] Mostafidi M., Sanjabi M.R., Shirkhan F., Zahedi M.T. (2020). A review of recent trends in the development of the microbial safety of fruits and vegetables. Trends Food Sci. Technol..

[14] Hounsou M., Dabadé D.S., Götz B., Hounhouigan M.H., Honfo F.G., Albrecht A., Dresch L.C., Kreyenschmidt J., Hounhouigan D.J. (2022). Development and use of food packaging from plant leaves in developing countries. Trends Food Sci. Technol..

[15] Shinde S.P., Hon G.R., P S., Chaudhari S.R., Matche R.S. (2024). Revamping ethylene absorption utilizing brick ash in packaging for prolonging the freshness of banana leaves. ACS Food Sci. Technol..

[16] Peng H., Simko I. (2023). Extending lettuce shelf life through integrated technologies. Curr. Opin. Biotechnol..

[17] Pignata G., Ertani A., Casale M., Piano S., Nicola S. (2020). Mixing fresh-cut baby green and red leaf lettuce from soilless cultivation preserves phytochemical content and safety. Agric. Food Sci..

[18] Steensma P., Widjaja F., Rajaraman S., Annala L., Klami A., Salojärvi J., Lehtonen M., Mikkonen K.S., Kangasjärvi S. (2025). Distinct post-harvest deterioration pathways caused by light vs dark storage of fresh-cut lettuce packed in modified atmosphere. Postharvest Biol. Technol.

[19] Nascimento L.B.D.S., Gori A., Cavigli L., Marino G., Brunetti C., Haworth M., Micheletti F., Pöhnl T., Neugart S., Agati G. (2024). UVB treatments of packaged ready-to-eat salads: Induced enhancement of quercetin derivatives in baby-leaf lettuce (*Lactuca sativa* L.) and wild rocket (*Diplotaxis tenuifolia* L.). Postharvest Biol. Technol.

[20] Kim M.J., Moon Y., Tou J.C., Mou B., Waterland N.L. (2016). Nutritional value, bioactive compounds and health benefits of lettuce (*Lactuca sativa* L.). J. Food Compost. Anal..

[21] More A.S., Ranadheera C.S., Fang Z., Zhang P., Warner R., Ajlouni S. (2022). Using biological metabolites as biomarkers to predict safety and quality of whole and minimally processed spinach. Food Chem..

[22] Martínez-Ispizua E., Calatayud Á., Marsal J.I., Cannata C., Basile F., Abdelkhalik A., Soler S., Valcárcel J.V., Martínez-Cuenca M.-R. (2022). The nutritional quality potential of microgreens, baby leaves, and adult lettuce: An underexploited nutraceutical source. Foods.

[23] Mir S.A., Shah M.A., Mir M.M. (2017). Microgreens: Production, shelf life, and bioactive components. Crit. Rev. Food Sci. Nutr..

[24] Yang X., Gil M.I., Yang Q., Tomás-Barberán F.A. (2022). Bioactive compounds in lettuce: Highlighting the benefits to human health and impacts of preharvest and postharvest practices. Compr. Rev. Food Sci. Food.

[25] (2003). Microbiology of Food and Animal Feeding Stuffs: Horizontal Method for the Enumeration of Microorganisms: Colony-count Technique at 30 °C.

[26] (2008). Microbiology of Food and Animal Feeding Stuffs: Horizontal Method for the Enumeration of Yeasts and Moulds.

[27] Lichtenthaler H., Wellburn A.R. (1985). Determination of total carotenoids and chlorophylls a and b of leaf in different solvents. Biochem. Soc. Trans..

[28] Benzie I.F., Strain J.J. (1996). The ferric reducing ability of plasma (FRAP) as a measure of “antioxidant power”: The FRAP assay. Anal. Biochem..

[29] Singleton V.L., Rossi J.A. (1965). Colorimetry of total phenolics with phosphomolybdic-phosphotungstic acid reagents. Am. J. Enol. Vitic..

[30] Kampfenkel K., Vanmontagu M., Inzé D. (1995). Extraction and determination of ascorbate and dehydroascorbate from plant tissue. Anal. Biochem..

[31] Fiore L., Cardarelli M., Lliuya J.C.L., Bonini P., Santelli P., Colla G. (2025). Nanobubble- and microbubble aeration affect leaf quality without changing yield of lettuce grown in floating systems. Horticulturae.

[32] Nicola S., Pignata G., Tibaldi G. (2018). The floating growing system can assure a low microbial contamination of baby leaf vegetables at harvest. Acta Hortic..

[33] Du M., Xiao Z., Luo Y. (2022). Advances and emerging trends in cultivation substrates for growing sprouts and microgreens toward safe and sustainable agriculture. Curr. Opin. Food Sci..

[34] Conte A., Conversa G., Scrocco C., Brescia I., Laverse J., Elia A., Del Nobile M.A. (2008). Influence of growing periods on the quality of baby spinach leaves at harvest and during storage as minimally processed produce. Postharvest Biol. Technol..

[35] Liu T., Jiao X., Yang S., Zhang Z., Ye X., Li J., Qi H., Hu X. (2020). Crosstalk between GABA and ALA to improve antioxidation and cell expansion of tomato seedling under cold stress. Environ. Exp. Bot..

[36] Mulaosmanovic E., Lindblom T.U.T., Windstam S.T., Bengtsson M., Rosberg A.K., Mogren L., Alsanius B.W. (2021). Processing of leafy vegetables matters: Damage and microbial community structure from field to bag. Food Control.

[37] Calonico C., Delfino V., Pesavento G., Mundo M., Nostro A.L.A. (2019). Microbiological quality of ready-to-eat salads from processing plant to the consumers. J. Food Nutr. Res..

[38] Hoffmann T.G., Ronzoni A.F., Silva D.L.D., Bertoli S.L., Souza C.K.D. (2021). Cooling kinetics and mass transfer in postharvest preservation of fresh fruits and vegetables under refrigerated conditions. Chem. Eng. Trans..

[39] Zhou B., Luo Y., Huang L., Fonseca J.M., Yan H., Huang J. (2022). Determining effects of temperature abuse timing on shelf life of RTE baby spinach through microbial growth models and its association with sensory quality. Food Control.

[40] Mudau A.R., Soundy P., Araya H.T., Mudau F.N. (2018). Influence of modified atmosphere packaging on postharvest quality of baby spinach (*Spinacia oleracea* L.) Leaves. Hortscience.

[41] Lourenco A.B., Casajús V., Ramos R., Massolo F., Salinas C., Civello P., Martínez G. (2024). Postharvest shelf life extension of minimally processed kale at ambient and refrigerated storage by use of modified atmosphere. Food Sci. Technol. Int..

[42] Mcgill J.N., Nelson A.I., Steinberg M.P. (2010). Effects of Modified Storage Atmospheres on Ascorbic Acid and Other Quality Characteristics of Spinach. J. Food Sci..

[43] Flores M., Amorós A., Escalona V.H. (2023). Changes in agronomic, antioxidant compounds, and morphology parameters of green and red lettuces (*Lactuca sativa* L.) by successive harvests and UV-B supplementation. Horticulturae.

