# Supplementary material for: Mixing Ratio and Packaging Amount Synergistically Improved Antioxidant Properties of Baby Lettuce (Lactuca sativa L.) and Spinach (Spinacia oleracea L.) Mixes
Source: Foods. 2026 Feb 1;15(3):499. doi: 10.3390/foods15030499 (PMC12896943; doi:10.3390/foods15030499)

Supplementary table

Table S1: Fresh weigh loss of lettuce and spinach mixes after 9 d storage at 4 ºC.

**Treatments**		Fresh weigh loss (%)	
Mixing ratio		d1	d9
	100LB	0.00	0.42
	75LB	0.00	0.28
	50LB	0.00	0.34
Packaging amount			
	250 F	0.00	0.29
	125 F	0.00	0.41
Mixing ratio × Packaging amount			
	100LB×250F	0.00	0.35
	100LB×125F	0.00	0.49
	75LB×250F	0.00	0.22
	75LB×125F	0.00	0.33
	50LB×250F	0.00	0.29
	50LB×125F	0.00	0.40
	Mean	0.00	0.37
	SE	0.00	0.15
Significance		ns	ns
	Mixing ratio	ns	ns
	Packaging amount	ns	ns
	Mixing ratio × Packaging amount	ns	ns

ns – not significant.

Figure S1. The standard curves of total antioxidant capacity, total phenolic compounds, and vitamin C.

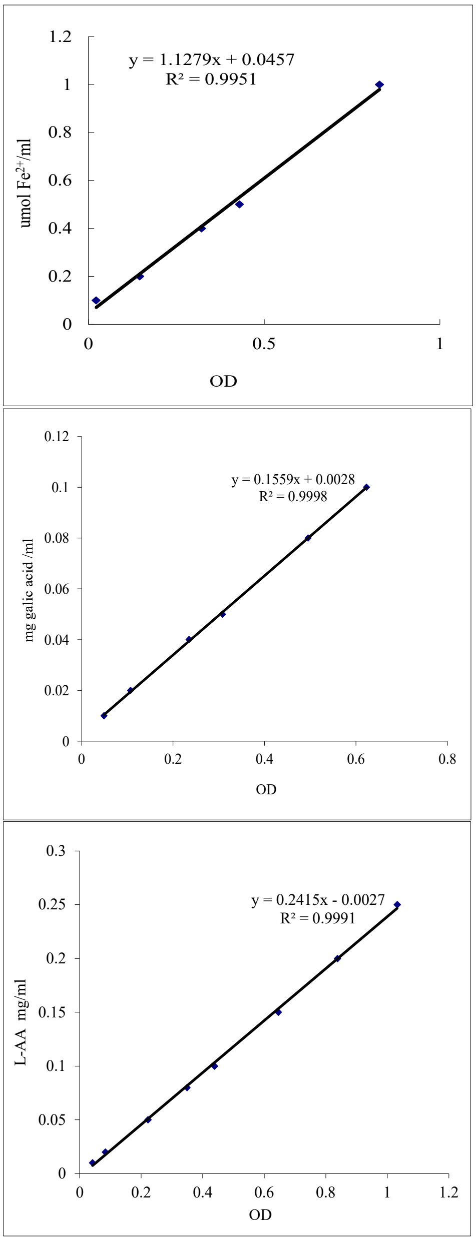

Supplement: Supplementary file 1 [file foods-15-00499-s001.zip › foods-4085623-supplementary.pdf]
